# Renal Manifestations of Tuberous Sclerosis Complex: Key Findings From the Final Analysis of the TOSCA Study Focussing Mainly on Renal Angiomyolipomas

**DOI:** 10.3389/fneur.2020.00972

**Published:** 2020-09-16

**Authors:** J. Chris Kingswood, Elena Belousova, Mirjana P. Benedik, Tom Carter, Vincent Cottin, Paolo Curatolo, Maria Dahlin, Lisa D'Amato, Guillaume Beaure d'Augères, Petrus J. de Vries, José C. Ferreira, Martha Feucht, Carla Fladrowski, Christoph Hertzberg, Sergiusz Jozwiak, John A. Lawson, Alfons Macaya, Ruben Marques, Rima Nabbout, Finbar O'Callaghan, Jiong Qin, Valentin Sander, Seema Shah, Yukitoshi Takahashi, Renaud Touraine, Sotiris Youroukos, Bernard Zonnenberg, Anna C. Jansen, Matthias Sauter

**Affiliations:** ^1^Cardiology Clinical Academic Group, Molecular and Clinical Sciences Research Centre, St Georges University of London, London, United Kingdom; ^2^Research and Clinical Institute of Pediatrics, Pirogov Russian National Research Medical University, Moscow, Russia; ^3^Child Neurology Department, SPS Pediatrična Klinika, Ljubljana, Slovenia; ^4^TSA Tuberous Sclerosis Association, Nottingham, United Kingdom; ^5^Hôpital Louis Pradel, Claude Bernard University Lyon 1, Lyon, France; ^6^Child Neurology and Psychiatry Unit, Systems Medicine Department, Tor Vergata University Hospital, Rome, Italy; ^7^Neuropediatric Department, Astrid Lindgren Children's Hospital, Stockholm, Sweden; ^8^Novartis Farma S.p.A., Origgio, Italy; ^9^Association Sclérose Tubéreuse de Bourneville, Gradignan, France; ^10^Division of Child and Adolescent Psychiatry, University of Cape Town, Cape Town, South Africa; ^11^Neurology Department, Centro Hospitalar Lisboa Ocidental, Lisbon, Portugal; ^12^Medical University of Vienna, Universitätsklinik für Kinder-und Jugendheilkunde, Affiliated Partner of the ERN EpiCARE, Vienna, Austria; ^13^Associazione Sclerosi Tuberosa ONLUS, Milan, Italy; ^14^European Tuberous Sclerosis Complex Association, Dattein, Germany; ^15^Zentrum für Neuropädiatrie und Sozialpädiatrie, Vivantes-Klinikum Neukölln, Berlin, Germany; ^16^Department of Child Neurology, Medical University of Warsaw, Warsaw, Poland; ^17^Department of Neurology and Epileptology, The Children's Memorial Health Institute, Warsaw, Poland; ^18^The Tuberous Sclerosis Multidisciplinary Management Clinic, Sydney Children's Hospital, Randwick, NSW, Australia; ^19^Pediatric Neurology Section, Hospital Universitari Vall d'Hebron, Barcelona, Spain; ^20^Institute of Biomedicine (IBIOMED), University of Leon, León, Spain; ^21^Department of Pediatric Neurology, Necker Enfants Malades Hospital, Paris Descartes University, Paris, France; ^22^Institute of Child Health, University College London, London, United Kingdom; ^23^Department of Pediatrics, Peking University People's Hospital (PKUPH), Beijing, China; ^24^Department of Neurology and Rehabilitation, Tallinn Children Hospital, Tallinn, Estonia; ^25^Novartis Healthcare Pvt. Ltd., Hyderabad, India; ^26^National Epilepsy Center, Shizuoka Institute of Epilepsy and Neurological Disorders, Shizuoka, Japan; ^27^Department of Genetics, CHU-Hôpital Nord, Saint-Étienne, France; ^28^First Department of Paediatrics, Athens University, St. Sophia Children's Hospital, Athens, Greece; ^29^Department of Internal Medicine, University Medical Center, Utrecht, Netherlands; ^30^Pediatric Neurology Unit, Department of Pediatrics, UZ Brussel VUB, Brussels, Belgium; ^31^Klinikverbund Allgäu gGmbH, Kempten, Germany

**Keywords:** mTOR, registry, renal angiomyolipoma, TOSCA, tuberous sclerosis complex

## Abstract

Renal angiomyolipomas are one of the most common renal manifestations in patients with tuberous sclerosis complex (TSC), with potentially life-threatening complications and a poor prognosis. Despite the considerable progress in understanding TSC-associated renal angiomyolipomas, there are no large scale real-world data. The aim of our present study was to describe in detail the prevalence and outcome of renal angiomyolipomas in patients with TSC, enrolled into the TuberOus SClerosis registry to increase disease Awareness (TOSCA) from 170 sites across 31 countries worldwide. We also sought to evaluate the relationship of TSC-associated renal angiomyolipomas with age, gender and genotype. The potential risk factors for renal angiomyolipoma-related bleeding and chronic kidney disease (CKD) were studied in patients who participated in the TOSCA renal angiomyolipoma substudy. Of the 2,211 eligible patients, 1,062 (48%) reported a history of renal angiomyolipomas. The median age of TSC diagnosis for the all subjects (*n* = 2,211) was 1 year. The median age of diagnosis of renal angiomyolipoma in the 1,062 patients was 13 years. Renal angiomyolipomas were significantly more prevalent in female patients (*p* < 0.0001). Rates of angiomyolipomas >3 cm (*p* = 0.0119), growing lesions (*p* = 0.0439), and interventions for angiomyolipomas (*p* = 0.0058) were also higher in females than males. Pre-emptive intervention for renal angiomyolipomas with embolisation, surgery, or mammalian target of rapamycin (mTOR) inhibitor may have abolished the gender difference in impaired renal function, hypertension, and other complications. The rate of interventions for angiomyolipomas was less common in children than in adults, but interventions were reported in all age groups. In the substudy of 76 patients the complication rate was too low to be useful in predicting risk for more severe CKD. In addition, in this substudy no patient had a renal hemorrhage after commencing on an mTOR inhibitor. Our findings confirmed that renal angiomyolipomas in subjects with *TSC1* mutations develop on average at the later age, are relatively smaller in size and less likely to be growing; however, by age 40 years, no difference was observed in the percentage of patients with *TSC1* and *TSC2* mutations needing intervention. The peak of appearance of new renal angiomyolipomas was observed in patients aged between 18 and 40 years, but, given that angiomyolipomas can occur later, lifelong surveillance is necessary. We found that pre-emptive intervention was dramatically successful in altering the outcome compared to historical controls; with high pre-emptive intervention rates but low rates of bleeding and other complications. This validates the policy of surveillance and pre-emptive intervention recommended by clinical guidelines.

## Introduction

Tuberous sclerosis complex (TSC) is a rare, autosomal dominant genetic disorder characterized by hamartomatous lesions in multiple organs such as brain, kidneys, skin, lungs, eyes, and heart (1, 2). Renal manifestations are one of the most common causes of morbidity and were historically reported as the primary cause of death in adult TSC patients (3–5). The relative importance of mechanisms postulated to lead to impaired renal function are unknown (6) but a major risk factor may be intervention for renal angiomyolipomas (7).

Renal angiomyolipomas are the most common renal manifestations in patients with TSC, with an estimated prevalence ranging from 55 to 80% (8–11). They are usually multiple and bilateral, progress with age and cause more problems in females (12, 13). Angiomyolipomas >3 cm in diameter have an increased risk of bleeding or invade adjacent normal renal parenchyma, potentially leading to kidney failure (10, 14). A retrospective cohort study showed that modifiable factors such as hypertension, proteinuria, and hyperfiltration occur frequently and early in patients with TSC and could play an important role in the development of chronic kidney disease (CKD) in these patients (15). Renal cysts, although asymptomatic in most patients, may be aggressive due to associated polycystic disease in a minority of patients and can even result in development of end stage renal disease in childhood or early adulthood (10, 16). Mutation studies have shown the occurrence and severity of TSC-associated renal angiomyolipomas and cysts to be higher among patients with *TSC2* mutation than those with *TSC1* mutation (8, 17).

Previously we have reported interim analysis data of the TOSCA (TuberOus SClerosis registry to increase disease Awareness) study, highlighting the burden of TSC-associated renal angiomyolipoma and showed that renal angiomyolipomas are initially asymptomatic, influenced by gender and genotype and can occur in younger patients (13). Here we present the final analysis data of the TOSCA registry with detailed overall characteristics of TSC-associated renal angiomyolipoma and its association with age, gender, and genotype. We have also analyzed possible risk factors for bleeding from renal angiomyolipomas and for CKD in patients with TSC from the TOSCA renal angiomyolipoma substudy.

## Materials and Methods

The study methodology has been published previously (18). In brief, TOSCA was a large-scale non-interventional study in patients with TSC. The study was designed with a core section and six ancillary substudies (research projects with more detailed focus on subependymal giant cell astrocytomas, renal angiomyolipoma, and lymphangioleiomyomatosis, genetics, TSC-associated neuropsychiatric disorder, epilepsy, and patient's quality of life). Here we present findings from the core study and renal angiomyolipoma substudy.

The TOSCA study was designed and conducted according to the Guidelines for Good Clinical Practice and ethical principles outlined in the Declaration of Helsinki. Written informed consent was obtained from all patients, parents, or guardians prior to enrolment with prior endorsement by the local human research ethics committee.

### Participants and Procedure

In the core study, patients of any age with TSC were enrolled from 170 sites across 31 countries and were followed for up to 5 years. Investigators from 18 sites across eight countries also agreed to participate in this renal angiomyolipoma substudy and enrolled a total of 76 patients, after receiving separate informed consent from the patients.

In the core study, patient data including demographics and clinical features of TSC across all organ systems, comorbidities, and rare manifestations, were collected at baseline and at regular visits scheduled at a maximum interval of 1 year. For the purpose of this manuscript, we presented data specific to renal angiomyolipoma including occurrence rate, annual incidence of newly diagnosed angiomyolipoma, maximum diameter on ultrasound or magnetic resonance imaging, clinical symptoms and complications, and management at baseline and during follow-up. The number of patients who completed follow-up 4 and follow-up 5 visits were low due to their late enrolment in the study, and hence follow-up data of only the first 3 years of the core study are reported here.

In the 76 patients in the renal substudy data was collected on; prevalence and size of renal angiomyolipomas and complication rates (including bleeding, hypertension, and CKD). We also present the effects of treatment with embolization or mammalian target of rapamycin (mTOR) inhibitors on the risk of renal impairment. For the substudy, only the baseline data are reported here, as very few patients had follow-up visits due to their late enrolment in the study.

### Data Analyses

All eligible patients enrolled in the TOSCA registry and renal angiomyolipoma substudy, without any major protocol deviations, were included in the analysis. Given that the study was observational in nature, results reported in this manuscript are primarily descriptive statistics. Continuous variables were evaluated quantitatively (e.g., frequency, mean, standard deviation, median, range), and categorical variables (e.g., presence/absence of a manifestation) were analysed in terms of frequency distribution at baseline and at follow-ups.

The Cochran–Mantel–Haenszel test was performed to evaluate the rates of renal angiomyolipomas stratified by age groups (<18 and ≥18 years), gender (male and female) and mutation (*TSC1* and *TSC2*). The exact binomial test was used to evaluate the difference between proportion of patients with renal angiomyolipomas and those received treatment among both genders, regardless of age, and genetic mutation. Furthermore, we evaluated reported association of angiomyolipoma-related variables at baseline visit (rates of angiomyolipomas, angiomyolipomas with lesion >3 cm, growing angiomyolipomas, treatment of angiomyolipomas and symptoms) by age (<18 vs. ≥18 years), gender (male vs. female) and mutation (*TSC1* vs. *TSC2*) using Chi-square test. Statistical significance was set at *p* < 0.05.

## Results

### Findings From the Core Study

A total of 2,214 patients were enrolled from 170 sites across 31 countries. Of these, data of 2,211 eligible patients were analysed. Data of three patients were excluded due to major protocol deviations. Most patients were enrolled at sites where the principal investigators were pediatric neurologists (53%) or neurologists (17%).

Baseline demographics and clinical characteristics are summarized in Table 1. There were more females (52.1%) than males (47.9%), the majority of patients were under the age of 18 years (61.6%) and the median age at consent for the study was 13 years. The median age at first TSC diagnosis was 1 year (mean 6.9 years, range: <1–69 years). Molecular genetic testing was performed in 1,011 patients (45.7%). Of these, 64.2% had a *TSC2* mutation and 18.9% *TSC1* mutation. In 14.6% of patients, no mutation was identified. Of the 1,011 tested patients, 663 (65.6%) had pathogenic mutation, 43 (4.3%) had a variant of unknown significance and 23 patients (2.3%) had both a pathogenic mutation and variant of unknown significance. In 282 patients, the pathogenicity of the mutation was not recorded. Prenatal diagnosis of TSC was reported in 154 patients (7%). Parents of 1,036 of 2,211 patients (56.3%) were evaluated for TSC. Of these, 180 (17.4%) had mother, 126 (12.2) had fathers and 4 (0.4%) had both parents diagnosed with TSC. A considerable proportion of patients (23.6%) had relatives affected with TSC and patients with relatives also enrolled in TOSCA (10.6%).

**Table 1:** Baseline patient demographics and clinical characteristics.

**Characteristic**	**All patients (*N* = 2,211)**	**Patients with renal angiomyolipoma (*N* = 1062)**
Patients by age at consent		
≤ 2 years	282 (12.8)	25 (2.4)
>2 to ≤ 5 years	301 (13.6)	76 (7.2)
>5 to ≤ 9 years	334 (15.1)	133 (12.5)
>9 to ≤ 14 years	307 (13.9)	164 (15.4)
>14 to <18 years	138 (6.2)	79 (7.4)
≥18 to ≤ 40 years	625 (28.3)	411 (38.7)
>40 years	224 (10.1)	174 (16.4)
Median (range) age at diagnosis of TSC,^a^ years	1.0 (<1–69)	1.0 (<1–67)
Gender		
Male	1,059 (47.9)	447 (42.1)
Female	1,152 (52.1)	615 (57.9)
Genetic molecular testing performed	1,011 (45.7)	525 (49.4)
Genetic testing results^b^^,^^c^		
No mutation identified	148 (14.6)	80 (15.2)
*TSC1* mutation	191 (18.9)	63 (12.0)
*TSC2* mutation	649 (64.2)	373 (71.0)
Both *TSC1* and *TSC2* mutations	5 (0.5)	2 (0.4)
Mutation variation type^c^		
Only pathogenic mutation	663 (65.6)	343 (65.3)
Only variant of unknown significance	43 (4.3)	23 (4.4)
Both	23 (2.3)	5 (1.0)
Time from TSC clinical diagnosis to molecular testing, months, mean (SD)	81.8 (116.58)	118.3 (133.4)
Patients with prenatal TSC diagnosis	154 (7.0)	53 (5.0)

*SD, standard deviation; TSC, tuberous sclerosis complex*.

*Values are expressed as n (%) unless otherwise specified*.

a *Data available for 2,174 patients (all patients) and 1050 patients (cohort with renal angiomyolipoma at baseline)*.

b *Genetic testing results were not available for 18 patients (all patients) and 7 patients (cohort with renal angiomyolipoma at baseline)*.

c *Percentages were calculated from number of patients with genetic molecular testing performed*.

### Clinical Characteristics of Renal Angiomyolipomas

**Figure 1:**
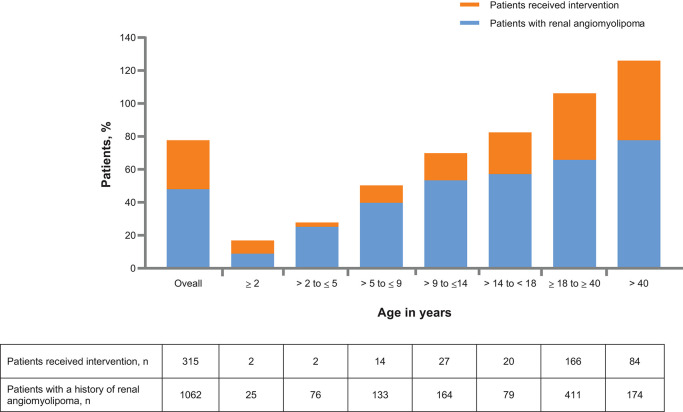
Patients with history of renal angiomyolipoma and intervention received across age groups at baseline.

A history of renal angiomyolipomas was reported in 1,062 (48%) patients (Table 2, Figure 1). Baseline demographics of cohort with renal angiomyolipomas were similar to the overall cohort (Table 1). Of 1,024 patients (96.4%) with ongoing renal angiomyolipoma, 901 (88%) had multiple lesions, 859 (83.9%) had bilateral lesions, 342 (33.4%) had lesions >3 cm in size and 216 (21.1%) had growing lesions. The median age at diagnosis was 13 years (mean 17 years, range <1–67 years). Median time from the previous scan to last assessment was 1 year (range, <1–21).

**Table 2:** Clinical characteristics of renal angiomyolipoma in overall population.

**Characteristic**	**Baseline *N* = 2,211**	**Follow-up 1*N* = 2,099**	**Follow-up 2 *N* = 1,935**	**Follow-up 3*N* = 1,664**
Past history of renal angiomyolipoma	1,062 (48.0)	–	–	–
Median (range) age at angiomyolipoma diagnosis, years	13 (<1–67)	–	–	–
Renal angiomyolipoma ongoing during the study^a^	1,024 (96.4)	1,024 (96.0)	1,002 (96.3)	909 (96.2)
Multiple	901 (88.0)	896 (87.5)	880 (87.8)	822 (90.4)
Bilateral	859 (83.9)	854 (83.4)	834 (83.2)	784 (86.2)
Lesion >3 cm	342 (33.4)	327 (31.9)	320 (31.9)	282 (31.0)
Growing	216 (21.1)	193 (18.8)	205 (20.5)	168 (18.5)
Renal angiomyolipoma symptoms and complications^b^				
None	840 (82.0)	894 (87.3)	885 (88.3)	816 (89.8)
Elevated blood pressure	58 (5.7)	48 (4.7)	42 (4.2)	38 (4.2)
Hematuria (blood in urine)	43 (4.2)	31 (3.0)	22 (2.2)	20 (2.2)
Hemorrhage	55 (5.4)	16 (1.6)	15 (1.5)	13 (1.4)
Impaired renal function	39 (3.8)	35 (3.4)	36 (3.6)	34 (3.7)
Pain	63 (6.2)	37 (3.6)	27 (2.7)	17 (1.9)
Other	30 (2.9)	13 (1.3)	16 (1.6)	12 (1.3)
Patients received treatment for angiomyolipoma^c^	315 (29.7)	300 (28.1)	321 (30.8)	288 (30.5)
mTOR inhibitor	144 (45.7)	49 (16.3)	28 (8.7)	26 (9.0)
Embolization	141 (44.8)	9 (3.0)	9 (2.8)	3 (1.0)
Nephrectomy	63 (20.0)	5 (1.7)	3 (0.9)	1 (0.3)
Resection	21 (6.7)	1 (0.3)	2 (0.6)	0
Dialysis	4 (1.3)	1 (0.3)	1 (0.3)	0
Other	13 (4.1)	1 (0.3)	5 (1.6)	1 (0.3)

*mTOR, mammalian target of rapamycin*.

*Values are expressed as n (%) unless otherwise specified*.

a *Percentages calculated based on denominator of patients with history of renal angiomyolipoma*.

b *Percentages calculated from number of patients with renal angiomyolipoma ongoing during the study*.

b *The numbers include patients who experienced more than one symptoms simultaneously*.

c *Treatment received as monotherapy or polytherapy*.

**Figure 2:**
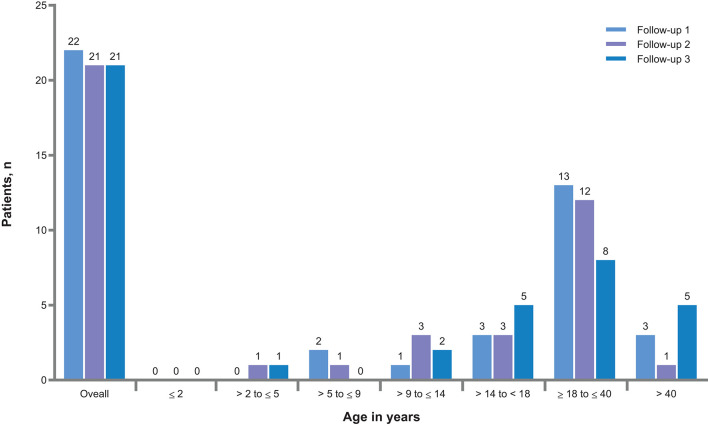
Newly diagnosed renal angiomyolipoma after baseline visit.

**Figure 3:**
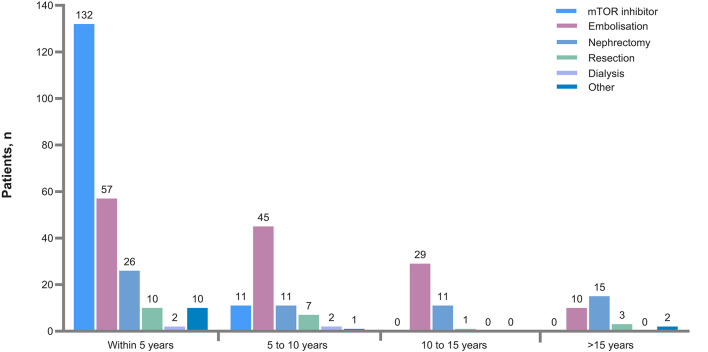
Time since interventions prior to baseline. mTOR, mammalian target or rapamycin.

Renal angiomyolipomas were asymptomatic in most patients (840 of 1,024 patients, 82%). Very few patients experienced renal angiomyolipoma-related symptoms or complications (Table 2). After baseline visit, newly diagnosed renal angiomyolipomas were reported in 22 (2.1%), 21 (2.0%), and 21 (2.2%) patients at follow-up 1, follow-up 2, and follow-up 3, respectively (Figure 2). A total of 315 patients (29.7%) had received treatment for renal angiomyolipomas at baseline. In these patients, mTOR inhibitors (45.7%), embolization (44.8%), and nephrectomies (20%) were the common treatment modalities. During the follow-ups, more patients received treatment with mTOR inhibitors than embolization (Table 2), and mTOR inhibitors appear to become a predominant treatment in recent years (Figure 3). However, the rate of nephrectomy was similar in each period prior to baseline.

### Relationship of Renal Angiomyolipoma With Age

**Figure 4:**
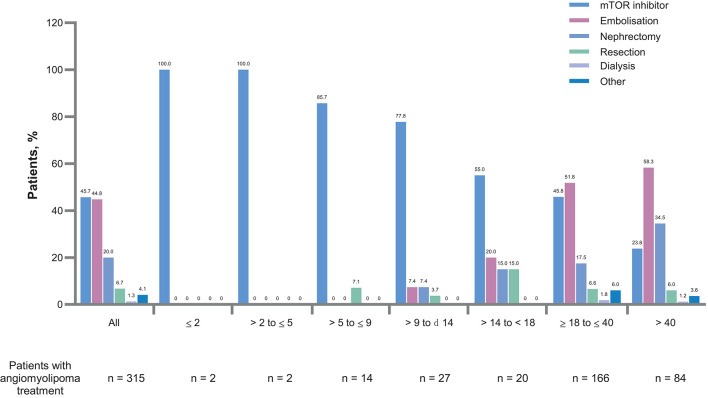
Treatment modalities for renal angiomyolipomas by age groups. mTOR, mammalian target or rapamycin.

The proportion of patients with angiomyolipomas increased with age (from 8.9% in patients aged ≤2 years to 77.7% in patients aged >40 years. Similarly, use of pre-emptive treatment increased with age (Figure 1). Newly diagnosed renal angiomyolipomas were more common in adults (Figure 2). There was an increased rate of symptoms and complications with age (Table 3). Embolization was more common in adults (54% vs. 9.2%), whereas children were mostly treated with mTOR inhibitors (73.8 vs. 38.4%), Figure 4).

**Table 3:** Renal angiomyolipoma symptoms and complications stratified by age.

**Complication and symptom**	**Overall (*N* = 2,211)**	**Age at consent, years**						
		**≤2 (*n* = 282)**	**>2 to ≤5 (*n* = 301)**	**>5 to ≤9 (*n* = 334)**	**>9 to ≤14 (*n* = 307)**	**>14 to <18 (*n* = 138)**	**≥18 to ≤40 (*n* = 625)**	**>40 (*n* = 224)**
None	840 (82.0)	23 (100.0)	74 (100.0)	122 (96.1)	147 (93.0)	71 (92.2)	298 (74.7)	105 (63.3)
Elevated blood pressure^a^	58 (5.7)	0 (0)	0 (0)	0 (0)	5 (3.2)	5 (6.5)	25 (6.3)	23 (13.9)
Hemorrhage^a^	43 (4.2)	0 (0)	0 (0)	0 (0)	2 (1.3)	1 (1.3)	23 (5.8)	17 (10.2)
Haematuria^a^	55 (5.4)	0 (0)	0 (0)	0 (0)	0 (0)	0 (0)	37 (9.3)	18 (10.8)
Impaired renal function^a^	39 (3.8)	0 (0)	0 (0)	1 (0.8)	2 (1.3)	0 (0)	16 (4.0)	20 (12.0)
Pain^a^	63 (6.2)	0 (0)	0 (0)	1 (0.8)	1 (0.6)	1 (1.3)	38 (9.5)	22 (13.3)
Other	30 (2.9)	0 (0)	0 (0)	3 (2.4)	2 (1.3)	1 (1.3)	17 (4.3)	7 (4.2)

*All the values are expressed as n (%)*.

a *The numbers include patients who experienced more than one symptom simultaneously*.

### Relationship of Renal Angiomyolipoma With Gender

Of the 2,211 enrolled patients, 1,152 (52.1%) were female and 1,059 (47.9%) were male. A history of renal angiomyolipomas was reported at a significantly higher frequency in female than male patients (53.4 vs. 42.2%, *p* < 0.0001, Table 4). Newly diagnosed renal angiomyolipomas were also more common in female patients (2.3 vs. 1.8%). The gender difference (female vs. male) in the rates of renal angiomyolipomas remained statistically significant when stratified by age [<18 years [38.97 vs. 31.54%]; *p* < 0.0001 and ≥18 years [71.35 vs. 65.18%]; *p* < 0.0001].

**Table 4:** Clinical characteristics of renal angiomyolipoma by gender.

**Characteristics**	**Female *N* = 1,152**	**Male*N* = 1,059**	**Odds ratio (95% CI)**	***P*-value**
Past history of renal angiomyolipoma	615 (53.4)	447 (42.2)	1.6 (1.3, 1.9)	<0.0001
Median (range) age at angiomyolipoma diagnosis, years	14 (<1–63)	11 (<1–67)	–	0.9891
Renal angiomyolipoma ongoing during the study^a^	590 (95.9)	434 (97.1)		
Multiple	524 (88.8)	377 (86.9)	1.2 (0.8, 1.8)	0.3436
Bilateral	502 (85.1)	357 (82.3)	1.3 (0.9, 1.8)	0.1585
Lesion >3 cm	212 (35.9)	130 (30.0)	1.4 (1.1, 1.9)	0.0119
Growing	135 (22.9)	81 (18.7)	1.4 (1.0, 1.9)	0.0439
Renal angiomyolipoma signs and symptoms^b^^,^^c^				
None	466 (79.0)	374 (86.2)	0.6 (0.4, 0.8)	0.0031
Elevated blood pressure	31 (5.3)	27 (6.2)	0.8 (0.5, 1.4)	0.5083
Haematuria (blood in urine)	29 (4.9)	14 (3.2)	1.6 (0.8, 3.0)	0.1829
Hemorrhage	41 (6.9)	14 (3.2)	2.2 (1.2, 4.2)	0.0090
Impaired renal function	27 (4.6)	12 (2.8)	1.7 (0.8, 3.4)	0.1345
Pain	50 (8.5)	13 (3.0)	3.0 (1.6, 5.6)	0.0003
Others	22 (3.7)	8 (1.8)	2.1 (0.9, 4.7)	0.0771
Treatment received for renal angiomyolipoma^d^	203 (33.0)	112 (25.1)		
mTOR inhibitor	95 (46.8)	49 (43.8)	1.1 (0.7, 1.78)	0.6395
Embolization	84 (41.4)	57 (50.9)	0.7 (0.4, 1.1)	0.0894
Nephrectomy	47 (23.2)	16 (14.3)	1.8 (1.0, 3.4)	0.0629
Resection	16 (7.9)	5 (4.5)	1.8 (0.6, 5.1)	0.2503
Dialysis	3 (1.5)	1 (0.9)	1.7 (0.2, 16.1)	0.6618
Other	10 (4.9)	3 (2.7)	1.9 (0.5, 6.9)	0.3428

*CI, confidence interval; mTOR, mammalian target of rapamycin*.

*Values are expressed as n (%) unless otherwise specified*.

a *Percentages calculated based on denominator of patients with history of renal angiomyolipoma*.

b *Percentages calculated from number of patients with renal angiomyolipoma ongoing during the study*.

c *The numbers include patients who experienced more than one symptom simultaneously*.

d *Treatment received as monotherapy or polytherapy*.

The median age at diagnosis of renal angiomyolipomas in female patients was 14 years (mean 18.4 years, range <1–63 years), while it was 11 years (mean 15.1 years, range <1–67 years) in male patients. The difference in the age at diagnosis between male and female patients were not significant (*p* = 0.9891). Five hundred and ninety females and 434 males had renal angiomyolipomas ongoing during the study. There was no significant differences between females and males in the occurrence of multiple lesions (88.8 vs. 86.9%, *p* = 0.3436) and bilateral angiomyolipomas (85.1 vs. 82.3%, *p* = 0.1585). Compared to males, females had significantly higher rates of lesions >3 cm in size (35.9 vs. 30.0%, *p* = 0.0119) and growing lesions (22.9 vs. 18.7%, *p* = 0.0439) at baseline. In both male and female patients, renal angiomyolipomas were asymptomatic in most patients at baseline (male: 86.2 vs. female: 79%). Most angiomyolipoma-related symptoms occurred equally in females and males. These include elevated blood pressure (5.3 vs. 6.2%, *p* = 0.5083), haematuria (4.9 vs. 3.2%, *p* = 0.1829) and impaired renal function (4.6 vs. 2.8%, *p* = 0.1345). However, compared to males, females had significantly higher rates of hemorrhage (6.9 vs. 3.2%, *p* = 0.0090) and pain (8.5 vs. 3%, *p* = 0.0003). Overall, the rate of intervention at baseline were significantly higher among females than males (33 vs. 25.1%, *p* = 0.0058). However, there was no significant gender difference (male vs. female) observed in the rates of specific interventions: embolization (50.9 vs. 41.4%; *p* = 0.0894), mTOR inhibitors (46.8 vs. 43.8%; *p* = 0.6395), nephrectomy (23.2 vs. 14.3%; *p* = 0.0629), resection (7.9 vs. 4.5%; *p* = 0.2503), and dialysis (1.5 vs. 0.9%; *p* = 0.6618).

### Relationship of Renal Angiomyolipoma With Mutation Type

The prevalence of angiomyolipomas was significantly higher in patients with *TSC2* vs. *TSC1* mutations (57.5 vs. 33%, *p* < 0.0001; Table 5). The mean age at diagnosis of renal angiomyolipomas was 13.3 years (median, 9 years, range <1–59 years) in patients with a *TSC2* mutations, while it was 22.5 years (median 21 years, range <1–60 years) in those with a *TSC1* mutations. Patients with *TSC2* mutations also had significantly higher rates of multiple angiomyolipomas (92.3 vs. 67.2, *p* < 0.0001), bilateral angiomyolipomas (87 vs. 47.5%, *p* < 0.0001) angiomyolipoma lesions >3 cm (31.2 vs. 11.5%, *p* = 0.0013) and growing angiomyolipomas (23.2 vs. 9.8%, *p* = 0.0150).

**Table 5:** Clinical characteristics of renal angiomyolipoma by mutational status.

**Characteristics**	**Patients with *TSC1* mutation *N* = 196**	**Patients with *TSC2* mutation*N* = 654**	**Odds ratio (95% CI)**	***p*-value**
Past history of renal angiomyolipoma	63 (33.0)	373 (57.5)	2.8 (2.0, 3.9)	<0.0001
Male	28 (44.4)	169 (45.3)	–	–
Female	35 (55.6)	204 (54.7)	–	–
Median (range) age at angiomyolipoma diagnosis, years	21 (<1–60)	9 (<1–59)	–	0.0035
Renal angiomyolipoma ongoing during the study^a^	61 (93.8)	362 (96.5)		
Multiple	41 (67.2)	334 (92.3)	6.1 (3.1, 11.8)	<0.0001
Bilateral	29 (47.5)	315 (87.0)	8.1 (4.4, 14.7)	<0.0001
Lesion >3 cm	7 (11.5)	113 (31.2)	3.6 (1.6, 8.2)	0.0013
Growing	7 (11.5)	85 (23.5)	2.9 (1.2, 7.2)	0.0150
Renal angiomyolipoma signs and symptoms^b^				
None	55 (90.2)	301 (83.1)	0.6 (0.2, 1.3)	0.1881
Elevated blood pressure	4 (6.6)	23 (6.4)	0.9 (0.3, 2.8)	0.9098
Haematuria (blood in urine)	0	14 (3.9)	NE	0.1234
Hemorrhage	0	19 (5.2)	NE	0.0709
Impaired renal function	1 (1.6)	10 (2.8)	1.7 (0.2, 13.2)	0.6297
Pain	2 (3.3)	24 (6.6)	2.0 (0.5, 8.8)	0.3335
Other	0	9 (2.5)	NE	0.2195
Treatment received for renal angiomyolipoma^a^^,^^c^	9 (13.8)	103 (27.5)	–	p < 0.0801
mTOR inhibitor	4 (44.4)	56 (54.4)	1.5 (0.4, 5.9)	0.5670
Embolization	2 (22.2)	41 (39.8)	2.3 (0.5, 11.7)	0.2983
Nephrectomy	3 (33.3)	23 (22.3)	0.6 (0.1, 2.5)	0.4534
Resection	1 (11.1)	6 (5.8)	0.5 (0.1, 4.6)	0.5299
Dialysis	0	1 (1.0)	NE (NE)	0.7665
Other	0	3 (2.9)	NE (NE)	0.6038

*CI, confidence interval; mTOR, mammalian target of rapamycin; TSC, tuberous sclerosis complex*.

*Values are expressed as n (%) unless otherwise specified*.

a *Percentages calculated based on denominator of patients with history of renal angiomyolipoma*.

b *Percentages calculated from number of patients with renal angiomyolipoma ongoing during the study*.

c *Treatment received as monotherapy or polytherapy*.

Similar to the overall sample, renal angiomyolipomas were asymptomatic in most patients with *TSC1* (90.2%) and *TSC2* (83.1%) mutations. However, bleeding events were observed only in patients with *TSC2* mutations (haematuria, 3.9% and hemorrhage, 5.2%). No significant difference in the rates of intervention of any sort was observed between those with *TSC1* mutations and *TSC2* mutations (*p* < 0.0801, Table 5).

### Other Renal Manifestations

The other renal features reported at baseline were multiple renal cysts (24.6%), polycystic kidney disease (proven TSC2/PKD1 mutation; 3.4%), renal malignancy (1.4%), and impaired renal function (non-angiomyolipoma-related; 1.9%) (Table 6). Compared with patients with a *TSC1* mutation, those with *TSC2* mutations had a higher occurrence of multiple renal cysts (33.6 vs. 13.3%) and polycystic kidney disease (4.7 vs. 0%).

**Table 6:** Rates of other renal manifestations at baseline in overall population and by mutational status.

	**Overall *N* = 2,211**	**Patients with *TSC1* mutation*N* = 196**	**Patients with *TSC2* mutation *N* = 654**
Renal manifestations in patients with angiomyolipomas			
Multiple renal cysts	544 (24.6)	26 (13.3)	220 (33.6)
Polycystic kidneys	Not applicable^*^	0	31 (4.7)
Renal malignancy	31 (1.4)	4 (2.0)	8 (1.2)
Renal manifestations in patients without angiomyolipoma			
Impaired renal function	43 (1.9)	6 (3.1)	18 (2.8)

*CI, confidence interval; mTOR, mammalian target of rapamycin; N/A, not applicable; TSC, tuberous sclerosis complex*.

*Values are expressed as n (%)*.

* *PKD was observed only in those with TSC2 mutations*.

### Findings From the Angiomyolipoma Substudy

A total of 76 patients [24 (31.6%) male and 52 (68.4%) female] were enrolled into the substudy from eight countries [France (*n* = 25), United Kingdom (*n*= 15), Belgium and Japan (*n* = 11, each), Turkey (*n* = 6), Poland (*n* = 4), and Germany and Spain (*n* = 2, each)]. Most patients were Caucasians (57 patients, 75%). Hypertension was reported in 19 patients (25%). Pre-existing antihypertensive medication was reported in 12 patients (63.2%).

### Risk Factors of Bleeding From Renal Angiomyolipomas

Of the 76 patients with renal angiomyolipomas, hemorrhage was reported in three patients at baseline, who were not taking mTOR inhibitors (patients aged 31, 34, and 43 years). All three of them were female and had *TSC2* mutations, with largest angiomyolipoma diameter between 66 and 96 mm.

### Risk Factors of Chronic Kidney Disease

A total of 42 patients reported CKD at baseline. Of these, seven (16.7%) had grade 3a/3b CKD (GFR 30–59), and four (9.5%) had grade 4 CKD (GFR 15–29). Thirty-six of 42 CKD patients had typical renal angiomyolipomas, eight had atypical renal angiomyolipomas and two had other renal angiomyolipomas. There was no correlation between CKD stage and type of angiomyolipoma. Mean age at diagnosis of renal angiomyolipoma was 14.5 years for patients with grade 1 CKD, 26.4 years for patients with grade 2 CKD, 35 years for patients with grade 3a CKD, 22 years for patients with grade 3b CKD and 34 years for patients with grade 4 CKD. Size of renal angiomyolipomas were between 3 and 180 mm. Simple cysts were reported in 16 patients (38.1%) and polycystic kidney disease in two patients (4.8%). Of the three patients with CKD and cysts, but without renal angiomyolipoma at baseline, two had grade 1 CKD and one had grade 2 CKD.

### Effect of Embolization or mTOR Inhibitor Treatment on CKD and Bleeding

Out of 76 patients enrolled, 47 patients received treatment; 20 were treated with mTOR inhibitors alone, four with embolization alone and five with both mTOR inhibitors and embolization at baseline. Among the 20 patients who were treated with mTOR inhibitors alone, eight (40%) had grade 2 CKD, four (20%) had grade 3a/3b CKD, and two had grade 4 CKD. No patient had unselected proteinuria while 7 patients (35%) had albuminuria grade 1. No patient on mTOR inhibitors alone had renal hemorrhage.

Among the four patients treated with embolization alone, one (25%) had grade 1 CKD, one (25%) had grade 2 CKD, and one (25%) had grade 4 CKD. Data was missing for one patient. One (25%) patient had proteinuria, while two (50%) had grade 1 albuminuria. No patient had renal hemorrhage.

## Discussion

The results from this final analysis have several novel observations. The prevalence of angiomyolipoma as well as rates of angiomyolipoma-related complications were higher in females than in male patients. This effect might be attributed to the presence of estrogen and progesterone receptors on the tumors (19). However, the mechanism of hormonal modulation on angiomyolipoma growth is not yet known. Female patients were also more likely to have bilateral, multiple and growing renal angiomyolipoma than male patients. This was in line with the other studies suggesting a higher propensity of angiomyolipoma growth in female patients (9, 20). Angiomyolipomas were dignosed at a later age in females (median age 14 years) than in male patients (median age 11 years), but this difference was not statistcally significant.

In our previous publication from the TOSCA core section interim analysis (13), we reported that the occurrence rate of renal angiomyolipomas was lower in the TOSCA cohort compared to other published literature (8, 9). Rates of haematuria and hypertension were also lower compared with those reported in TSC patients in other studies (6, 7, 21, 22), this may be a reflection of the age relatively young age of our subjects and possibly under-ascertainment. These lower rates of occurrence of renal angiomyolipomas and angiomyolipoma-related complications could be explained by a different (younger) age range of our population; however the current analysis shows that angiomyolipoma prevalence rose progressively with age, to 77.7% in those over 40 years of age, whereas complication rates remained much lower than in other studies. This suggests that active surveillance and a policy of pre-emptive treatment may have been successful in altering the natural history of renal TSC.

Patients with *TSC2* mutations were reported to exhibit a higher incidence and severity of both renal angiomyolipoma and cysts than those with *TSC1* mutations (8). In our study, the prevalence of angiomyolipoma was significantly higher in those with *TSC2* mutations. This was in line with the previous other reports (7, 8, 17, 23). We also observed that patients with *TSC2* mutations had angiomyolipoma at early age and experienced higher rates of bleeding complications (haematuria and hemorrhage). Rates of multiple angiomyolipomas, bilateral angiomyolipoma, renal angiomyolipoma lesions of >3 cm were significantly higher in those with *TSC2* mutations than those with *TSC1* mutations. Furthermore, more patients with *TSC2* mutations received intervention for renal angiomyolipoma than those with *TSC1* mutations.

As expected polycystic kidney disease was only found in those with *TSC2* mutations because it is the result of a deletion stretching across the TSC2 and PKD1 genes on chromosme 16 (The “contiguous gene syndrome”) (24).

The study showed that pre-emptive treatment was used increasingly commonly with age (Figure 1) and this was associated with a very low rate of bleeding and significant renal impairment. Figures 3, 4 show that mTOR inhibitors are now the most commonly used treatment.

Despite the fact that overall prevalence of hemorrhage and CKD was too low to accurately define risk factors, in our sub-study we observed that all the three patients who had hemorrhage had *TSC2* mutation. Majority of the patients had grade 1/2 CKD (31 patients, 73.8%). Patients with CKD grade 2 or more were older but there was a clear trend for more advanced CKD stages.

Renal malignancy has been reported in about 2–4% of patients with TSC (25), which is much higher than that reported in a comparable age group in the general population (26). The occurrence rate of renal malignancy observed in this cohort was lower (1.4%) than that reported previously, in TSC (8, 25).

## Conclusion

Renal angiomyolipomas are the major kidney risk for those with TSC; other renal complications are less common. We have shown a marked increase in the prevalence of intervention for renal angiomyolipomas, from <10% in those under 2 years of age to 48% in those over 40. The risk of needing an intervention was higher and begins earlier in those with a *TSC2* mutation, but the difference disappears by age 40 years. Gender differences were much smaller, but in females the occurrence of angiomyolipomas was significantly greater, as were angiomyolipomas >3 cm and the need for intervention. However, there was no absolute cut-off between the differences in any of these categories which means lifelong surveillance is important in all patients. In the substudy of 76 subjects none had a renal hemorrhage after commencing on an mTOR inhibitor. The most encouraging finding was that pre-emptive intervention was dramatically successful in altering the outcome compared to historical controls; with high pre-emptive intervention rates but low rates of bleeding and other complications. This validates the policy of surveillance and pre-emptive intervention recommended by clinical guidelines.

## Data Availability Statement

The raw data supporting the conclusions of this article will be made available by the authors, without undue reservation.

## List of Ethics Committees

The study protocol and all amendments were reviewed and approved (if applicable) by independent ethics committee/institutional review board for each centre: National Hospital Organization Central Ethics Committee; Gazi University Clinical Research Ethics Committee; Independent Multidisciplinary Committee on Ethical Review of Clinical Trials; Peking Union Medical College Hospital; Commissie Medische Ethiek UZ Brussel; CNIL (Commission National de l'Informatique et des Libertés), CCTIRS (Comité Consultatif sur le traitement de l'information en matière de recherche dans le domaine de la santé); Comité Etico Investigación Clínica de Euskadi (CEIC-E); Consejeria de Salud y Bienestar Social, Dirección General de Calidad, Investigación, Desarrollo e Innovación, Comité Coordinador de Ética de la Investigación Biomédica de Andalucía; Research Ethics Committee of the University of Tartu (UT REC); Ethikkommission der Medizinischen Universität Graz; North Wales REC—West; Regionala Etikprövningsnämnden i Göteborg; REK—Regionale komiteer for medisinsk og helsefaglig forskningsetikk; Komisja Bioetyczna przy Instytucie Pomnik Centrum Zdrowia Dziecka; Ethikkommission bei der Ludwig-Maximilians-Universitat München; Hokkaido University Hospital Independent clinical research Institutional Ethics Committee; Medical Juntendo University Institutional Ethics Committee; National Center for Chile Health and Deveropment of IRB; Osaka University Hospital of IRB; Ethics Committee at Moscow Institute of Pediatrics and Pediatric Surgery; Peking University First Hospital; Sanbo Brain Hospital Capital Medical University; Tianjin Children's Hospital; Children's Hospital of Fudan University; Zhongshan Hospital Fudan University; Fudan University Shanghai Cancer Center; The Second Affiliated Hospital of Guangzhou Medical University; The First Affiliated Hospital, Sun Yan-sen University; The First Affiliated Hospital of Guangzhou Medical University; Shenzhen Children's Hospital; West China Hospital, Sichuan University; Xijing Hospital; Children's Hospital of Chongqing Medical University; Wuhan Children's Hospital; The Second Affiliated Hospital of Xi'an Jiaotong university; Guangdong 999 Brain Hospital; Seoul National University Hospital Institutional Review Board; National Taiwan University Hospital (NTUH) Research Ethics Committee (REC); Institutional Review Board of the Taichung Veterans General Hospital; Institutional Review Board of Chung Shan Medical University Hospital; Institutional Review Board, Tungs' Taichung MetroHarbor Hospital; Institutional Review Board of National Cheng Kung University Hospital; Metro South Human Research Ethics Committee; Sydney Children's Hospital Network Human Research Ethics Committee; St Vincent's Hospital Human Research Ethics Committee; Royal Melbourne Hospital Human Research Ethics Committee; Siriraj Institutional Review Board; The Institutional Review board, Faculty of Medicine, Chulalongkorn University, 3rd Floor, Ananthamahidol Building, King Chulalongkorn Memorial Hospital; The Committee on Human Rights Related to Research Involving Human Subjects; Institutional Review Board, Royal Thai Army Medical Department IRB RTA, 5th Floor, Phramongkutklaowejvitya Building, Phramongkutklao College of Medicine; Research Ethics Committee, Faculty of Medicine, Chiang Mai University; Research and Development, Queen Sirikit National Institute of Child Health; Human Research Ethics Committee, Faculty of Health Sciences, University of Cape Town; Shaare Zedek Medical Center Helsinki Committee; Sheba Medical Center Helsinki Committee; Tel Aviv Sourasly Medical Center Helsinki Committee; General University Hospital of Patras Ethics Committee; Pendeli Children's Hospital Ethics Committee; General University Hospital of Athens G. Gennimatas Ethics Committee; Evaggelismos General Hospital Ethics Committee; General University Hospital of Thessaloniki AHEPA Ethics Committee; General University Hospital of Ionnina Ethics Committee; METC UMC Utrecht; Direcció General de Regulació, Planificació i Recursos Sanitaris; Comité Ético de Investigación Clínica del Hospital Universitario Vall d'Hebron de Barcelona, Generalitat de Catalunya. Departament de Salut; Comité Ético de Investigación Clínica Hospital Universitario La Paz; Dirección General de Ordenación e Inspección, Consejería de Sanidad Comunidad de Madrid, Servicios de Control Farmacéutico y Productos Sanitarios; Comité Etico Investigación Clínica del Hospital Universitario y Politécnico de La Fe; Dirección General de Farmàcia i Productes Sanitaris, Generalitat de Valencia; Comité de Ética de la Investigación de Centro de Granada; Instituto Aragonés de Ciencias de la Salud (IACS); Comité Etico Investigación Clínica Regional del Principado de Asturias; Comité Etico Investigación Clínica Hospital 12 de Octubre;, Comité Etico Investigación Clínica Hospital Universitario Virgen de la Arrixaca; Sección de Ordenación e Inspección Farmacéutica Departamento de Salud; Comité Ético de Investigación Clínica del Hospital Universitario del Río Hortega de Valladolid; Comissão de Ética para a Saúde (CES), Centro Hospitalar de Lisboa Ocidental, EPE; Comissão de Ética para a Saúde (CES), Centro Hospitalar do Porto, E.P.E; Comissão de Ética para a Saúde (CES), Centro Hospitalar Lisboa Central, EPE; Comissão de Ética para a Saúde (CES), Hospital Garcia de Orta, EPE; Comissão de Ética para a Saúde (CES), Centro Hospitalar de São João, EPE; Comissão de Ética para a Saúde (CES), Hospital Professor Doutor Fernando Fonseca, EPE; Comissão de Ética para a Saúde (CES), Centro Hospitalar do Algarve, EPE (Unidade de Faro); LUHS Kaunas Regional Biomedical Research Ethics Committee; Paula Stradina kliniskās universitātes slimnicas, Attistibas biedribas Kliniskās izpētes Etikas komiteja, Ethics Committee for Clinical Research; Komisija Republike Slovenije za medicinsko etiko; Comitato Etico Indipendente Presso La Fondazione Ptv Policlinico Tor Vergata Di Roma; Comitato Etico Regione Calabria Sezione Centro c/o A.O.U. Mater Domini Di Catanzaro; Comitato Etico Azienda Ospedaliera Universitaria Di Cagliari; Comitato Etico Cardarelli-Santobono c/o Ao Cardarelli; Comitato Etico Per La Sperimentazione Clinica Delle Province Di Verona E Rovigo, Presso Aoui Verona; Eticka Komise Fn Brno; Eticka Komisia Dfnsp Bratislava; Eticka Komisia Pri Dfn Kosice; Eticka Komisia Bratislavskeho Samospravneho Kraja; Comisia Naţională de Bioetică a Medicamentului şi a Dispozitivelor Medicale; Comitato Etico Milano area 1 c/o ASST FBF Sacco—P.O. L. Sacco; Comité de Ética de la Investigación de Centro Hospital Universitario Virgen del Rocío; Comité Ético de Investigación Clínica Fundació Sant Joan de Déu Generalitat de Catalunya. Departament de Salut; Comité Ético de Investigación Clínica Hospital Infantil Universitario Niño Jesús; Consejería de Sanidad Dirección General de Salus Pública Junta de Castilla León; Dirección General de Asistencia Sanitaria, Consejería de Sanidad Gobierno del Principado de Asturias; Dirección General de Planificación, Ordenación Sanitaria y Farmacéutica e Investigación, Consejeria de Sanidad y Política Social Región de Murcia; Ethics Committee at Moscow Institute of Pediatrics and Pediatric Surgery; Paula Stradina kliniskās universitātes slimnicas, Attistibas biedribas Kliniskās izpētes Etikas komiteja, Ethics Committee for Clinical Research; The First Affiliated Hospital of the Fourth Military Medical University; Zhongshan Hospital Fudan University.

## Ethics Statement

The studies involving human participants were reviewed and approved by all ethics committees involved in the TOSCA study (see list of ethics committees in article). Written informed consent to participate in this study was provided by the participants' legal guardian/next of kin.

## Author Contributions

JK, EB, MB, PC, MD, JF, MF, CH, SJ, JL, AM, RN, VS, RT, BZ, AJ, and MS designed the study, patient accrual, clinical care, data interpretation, drafted, revised, final review, and approval of the manuscript. TC, VC, GB, PV, CF, FO'C, JQ, YT, and SY designed the study, data interpretation, drafted, revised, final review, and approval of the manuscript. LD'A designed the study, trial management, data collection, data analysis, data interpretation, drafted, revised, final review, and approval of the manuscript. RM designed the study, data analysis, data interpretation, drafting, revised, final review, and approval of the manuscript. SS designed the study, trial statistician, data analysis, data interpretation, drafted, revising, final review, and approval of the manuscript. All authors contributed to the article and approved the submitted version.

## TOSCA Investigators

Japan: Nobuo Shinohara, Shigeo Horie, Masaya Kubota, Jun Tohyama, Katsumi Imai, Mari Kaneda, Hideo Kaneko, Yasushi Uchida, Tomoko Kirino, Shoichi Endo, Yoshikazu Inoue, Katsuhisa Uruno; Turkey: Ayse Serdaroglu, Zuhal Yapici, Banu Anlar, Sakir Altunbasak; Russia: Olga Lvova, Oleg Valeryevich Belyaev, Oleg Agranovich, Elena Vladislavovna Levitina, Yulia Vladimirovna Maksimova, Antonina Karas; China: Yuwu Jiang, Liping Zou, Kaifeng Xu, Yushi Zhang, Guoming Luan, Yuqin Zhang, Yi Wang, Meiling Jin, Dingwei Ye, Weiping Liao, Liemin Zhou, Jie Liu, Jianxiang Liao, Bo Yan, Yanchun Deng, Li Jiang, Zhisheng Liu, Shaoping Huang, Hua Li; Korea: Kijoong Kim; Taiwan: Pei-Lung Chen, Hsiu-Fen Lee, Jeng-Dau Tsai, Ching-Shiang Chi, Chao-Ching Huang; Australia: Kate Riney, Deborah Yates, Patrick Kwan; Thailand: Surachai Likasitwattanakul, Charcrin Nabangchang, Lunliya Thampratankul Krisnachai Chomtho, Kamornwan Katanyuwong, Somjit Sriudomkajorn; South Africa: Jo Wilmshurst; Israel: Reeval Segel, Tal Gilboa, Michal Tzadok, Aviva Fattal- Valevski; Greece: Panagiotis Papathanasopoulos, Antigone Syrigou Papavasiliou, Stylianos Giannakodimos, Stylianos Gatzonis, Evangelos Pavlou, Meropi Tzoufi; Netherlands: A.M.H. Vergeer; Belgium: Marc Dhooghe, Hélène Verhelst, Filip Roelens, Marie Cecile Nassogne, Pierre Defresne, Liesbeth De Waele, Patricia Leroy, Nathalie Demonceau, Benjamin Legros, Patrick Van Bogaert, Berten Ceulemans, Lina Dom; France: Pierre Castelnau, Anne De Saint Martin, Audrey Riquet, Mathieu Milh, Claude Cances, Jean-Michel Pedespan, Dorothee Ville, Agathe Roubertie, Stéphane Auvin, Patrick Berquin, Christian Richelme, Catherine Allaire, Sophie Gueden, Sylvie Nguyen The Tich, Bertrand Godet; Spain: Maria Luz Ruiz Falco Rojas, Jaume Campistol Planas, Antonio Martinez Bermejo, Patricia Smeyers Dura, Susana Roldan Aparicio, Maria Jesus Martinez Gonzalez, Javier Lopez Pison, Manuel Oscar Blanco Barca, Eduardo Lopez Laso, Olga Alonso Luengo, Francisco Javier Aguirre Rodriguez, Ignacio Malaga Dieguez, Ana Camacho Salas, Itxaso Marti Carrera, Eduardo Martinez Salcedo, Maria Eugenia Yoldi Petri, Ramon Cancho Candela; Portugal: Ines da Conceicao Carrilho, Jose Pedro Vieira, José Paulo da Silva Oliveira Monteiro, Miguel Jorge Santos de Oliveira Ferreira Leao, Catarina Sofia Marceano Ribeiro Luis, Carla Pires Mendonca; Lithuania: Milda Endziniene; Latvia: Jurgis Strautmanis; Estonia: Inga Talvik; Italy: Maria Paola Canevini, Antonio Gambardella, Dario Pruna, Salvatore Buono, Elena Fontana, Bernardo Dalla Bernardina; Romania: Carmen Burloiu, Iuliu Stefan Bacos Cosma, Mihaela Adela Vintan, Laura Popescu; Czech Republic: Karel Zitterbart; Slovakia: Jaroslava Payerova, Ladislav Bratsky, Zuzana Zilinska; Austria: Ursula Gruber-Sedlmayr, Matthias Baumann, Edda Haberlandt, Kevin Rostasy, Ekaterina Pataraia; United Kingdom: Frances Elmslie, Clare Ann Johnston, Pamela Crawford; Denmark: Peter Uldall; Sweden: Paul Uvebrant, Olof Rask; Norway: Marit Bjoernvold, Eylert Brodtkorb, Andreas Sloerdahl, Ragnar Solhoff, Martine Sofie Gilje Jaatun; Poland: Marek Mandera, Elzbieta Janina Radzikowska, Mariusz Wysocki; Germany: Michael Fischereder, Gerhard Kurlemann, Bernd Wilken, Adelheid Wiemer-Kruel, Klemens Budde, Klaus Marquard, Markus Knuf, Andreas Hahn, Hans Hartmann, Andreas Merkenschlager, Regina Trollmann.

## Conflict of Interest

JK, EB, TC, VC, PC, GB, JF, PV, MF, CF, CH, SJ, RN, FO'C, JQ, MS, RT, MD, JL, AM, SY, MB, BZ, and AJ received honoraria and support for travel from Novartis. VC received personal fees for consulting, lecture fees and travel from Actelion, Bayer, Biogen Idec, Boehringer Ingelheim, Gilead, GSK, MSD, Novartis, Pfizer, Roche, and Sanofi; grants from Actelion, Boehringer Ingelheim, GSK, Pfizer, and Roche; personal fees for developing educational material from Boehringer Ingelheim, and Roche. PV has been on the study steering group of the EXIST-1, 2, and 3 studies sponsored by Novartis and co-PI on two investigator-initiated studies part-funded by Novartis. RN received grant support, paid to her institution, from Eisai and lectures fees from Nutricia, Eisai, Advienne, and GW Pharma. YT received personal fee from Novartis for lecture and for copyright of referential figures from the journals and received grant from Japanese government for intractable epilepsy research. SJ was partly financed by the EC Seventh Framework Programme (FP7/2007-2013; EPISTOP, grant agreement No. 602391), the Polish Ministerial funds for science (years 2013–2018) for implementation of international co-financed project and the grant EPIMARKER of the Polish National Center for Research and Development No. STRATEGMED3/306306/4/2016. JK, PC, CH, JL, and JQ received research grant from Novartis. RM and SS are employees of Novartis, while LD'A was a Novartis employee at the time of manuscript concept approval. The remaining authors declare that the research was conducted in the absence of any commercial or financial relationships that could be construed as a potential conflict of interest.
